# 1-hour versus 3-hour ^99m^Tc-PYP imaging to evaluate suspected cardiac transthyretin amyloidosis

**DOI:** 10.1097/MD.0000000000033817

**Published:** 2023-05-17

**Authors:** Kenneth J. Nichols, Se-Young Yoon, Andrew Van Tosh, Christopher J. Palestro

**Affiliations:** a Department of Radiology, Donald and Barbara Zucker School of Medicine at Hofstra/Northwell, Hempstead, NY; b Radiology, Beth Israel Deaconess Medical Center/Harvard Medical School, Boston, MA; c St. Francis Hospital, Roslyn, NY.

**Keywords:** amyloid heart disease, hybrid imaging, image interpretation, SPECT/CT

## Abstract

The diagnosis of cardiac transthyretin amyloidosis can involve early or delayed ^99m^Tc-pyrophosphate planar, single photon emission computed tomography (SPECT), and/or SPECT/CT imaging. We investigated whether image interpretations differed among modalities and time points. In this observational study, data were reviewed for 173 patients with suspected transthyretin amyloidosis who underwent planar and SPECT/CT 1 and 3 hours after radiopharmaceutical injection. Planar heart-to-contralateral lung ratios were calculated. Myocardial-to-rib uptake was independently scored on SPECT and SPECT/CT as follows: 0 (negative), 1 < rib (equivocal), 2 = rib (positive), or 3 > rib (positive), and the image quality was as follows:1 (poor), 2 (adequate), and 3 (good). Three-hour SPECT/CT readings were used as the reference standard against which the other readings were compared. Twenty-five percent of patients were positive (3-hour SPECT/CT score ≥ 2). Compared to 3-hour SPECT/CT readings, there was “fair agreement” (*κ* = .27 − .33) with SPECT, and “fair agreement” (*κ* = .23 − .31) with planar imaging at 1 and 3 hours. More patients had abnormal SPECT and SPECT/CT than planar imaging (24–25% vs 16–17%, *P* < .007). There were more equivocal cases for 1 and 3 hours planar imaging than for 1 and 3 hours SPECT (71–73% vs 23–26%, *P* < .001) and 1 and 3 hours SPECT/CT (3–5%, *P* < .001). SPECT/CT image quality was higher at 3 hours than at 1 hour and higher than that on SPECT (*P* = .001). Three-hour SPECT/CT readings provided the highest number of definitive readings, had the highest image quality, and constituted the preferred protocol for evaluating unselected populations of patients that have a clinical suspicion of possible cardiac amyloidosis.

## 1. Introduction

^99m^Tc-pyrophosphate (PYP) imaging accurately diagnoses cardiac transthyretin amyloidosis (ATTR)^[1]^ and has been used to predict the survival of patients undergoing treatment for amyloidosis.^[2]^ Initial imaging guidelines recommend computing anterior view planar heart-to-contralateral lung (HCL) ratios obtained 1 hour post-injection.^[3,4]^ More recent recommendations consider planar imaging alone to be inadequate, particularly given the preponderance of equivocal results.^[5]^ Qualitative grading of single photon emission computed tomography (SPECT) or SPECT/CT 2 to 3 hours after radiopharmaceutical administration,^[6]^ which has been reported to be highly reproducible,^[7]^ currently is considered the standard of care.

There are competing criteria to consider when adopting imaging protocols. Planar imaging has the advantage of requiring the least expensive nuclear imaging equipment, being the least technically demanding, and requiring the least amount of time. SPECT is expected to provide better separation of the myocardium from the blood pool than planar imaging, and SPECT/CT should be superior to both planar imaging and SPECT for localizing activity. However, on a patient-by-patient basis, are the diagnoses produced by these 3 nuclear imaging modalities significantly different? It is expected that there will be more washout of blood pool activity at 3 hours than at 1 hour, providing a stronger visual target-to-background count ratio to facilitate the identification of foci of amyloid deposition at a later time point. However, is the magnitude of blood pool washout sufficiently great to justify the added inconvenience to patients and the reduced laboratory throughput efficiency necessitated by waiting 3 hours after radiopharmaceutical administration? Conversely, is washout so great at 3 hours that delaying planar imaging to 3 hours provides the same clarity for diagnosis as SPECT or SPECT/CT at 3 hours?

We performed this investigation to determine whether patients were classified statistically similarly at 1 and 3 hours for planar imaging, SPECT, and SPECT/CT and whether planar and SPECT readings were statistically similar to SPECT/CT readings. The reference standard for our study was the SPECT/CT results at 3 hours.

## 2. Methods and materials

### 2.1. Study design and eligibility criteria of patients

This was an observational retrospective investigation of data acquired at a single site between January 2018 and September 2021 in an unselected population of all patients with clinically suspected ATTR cardiac amyloidosis. The patient protocol conformed to standard imaging guidelines at that time, which stipulated an initial planar image of the chest at 1 hour, with or without additional SPECT, followed by repeat imaging at 3 hours.^[3]^ Patients were included for analysis only if they had both planar and SPECT/CT at both 1 and 3 hours following radiopharmaceutical injection; thus, there were data for a total of 173 patients available for analysis (108 male, 65 female, 72 ± 12 years).

The Northwell Institutional Review Board approved this retrospective study and waived the requirement for informed consent. All the data were handled in compliance with the Health Insurance Portability and Accountability Act of 1996.

### 2.2. Imaging

All patients underwent planar imaging and SPECT/CT 1 and 3 hours after intravenous injection of 555 MBq ^99m^Tc-PYP. Images were acquired using a dual Anger detector large field-of-view rotating SPECT/CT system equipped with low-energy high-resolution collimators and an integrated 6-slice diagnostic-quality CT unit (Symbia Intevo, Siemens Medical Solutions AG, Erlangen, Germany). The primary energy peak was centered at 140 keV (15% energy window) and the scatter correction peak was centered at 119 keV (18% energy window).

**Figure 1.**
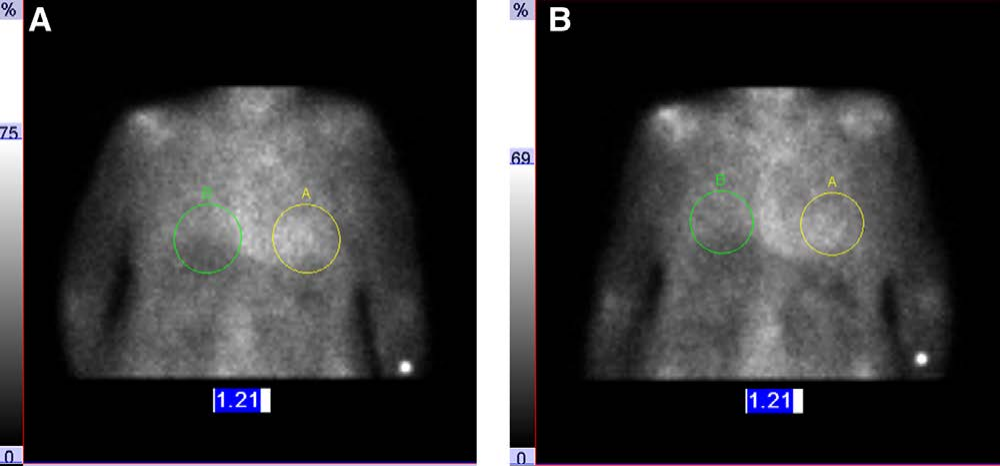
Planar images for the same patient acquired at (A) 1 h following tracer injection and (B) 3 h after radiopharmaceutical injection. Both planar imaging results were “equivocal,” as both had heart-to-contralateral lung count ratios of 1.21.

**Figure 2.**
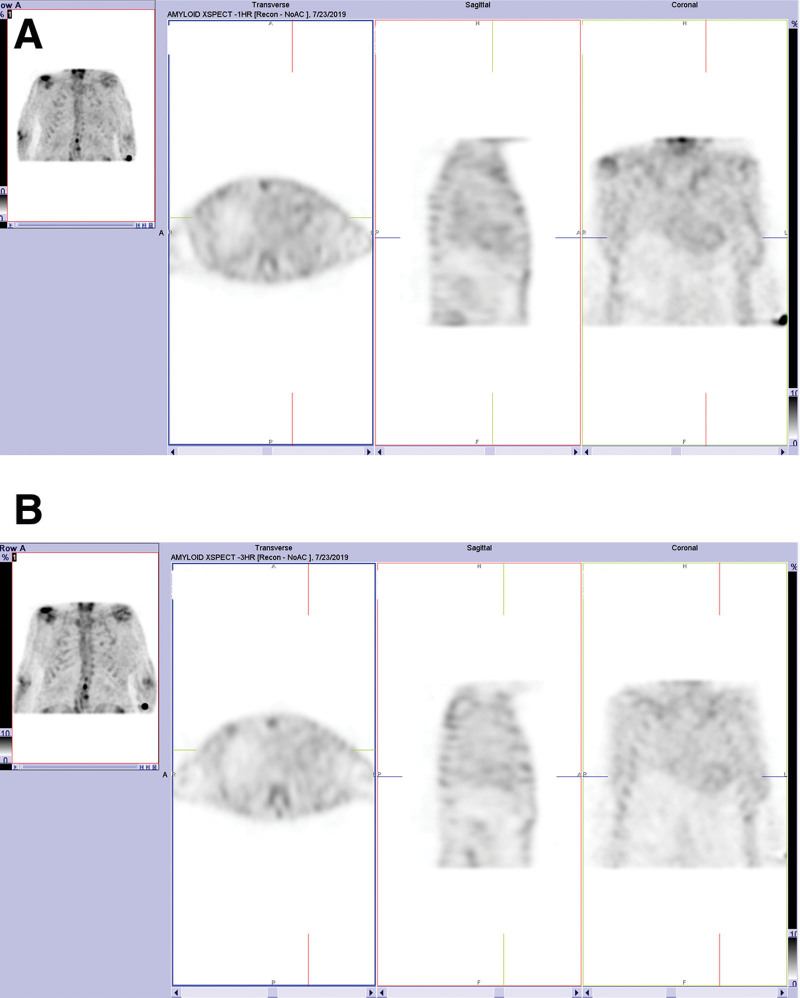
Uncorrected SPECT reconstructions for the same patient as Figure 1 for data acquired (A) 1 h and (B) 3 h following radiopharmaceutical injection. Both scans were scored as “1,” as the physician reading these studies perceived myocardial uptake to be less than that of rib uptake and not convincingly higher than blood pool activity. SPECT = single photon emission computed tomography.

**Figure 3.**
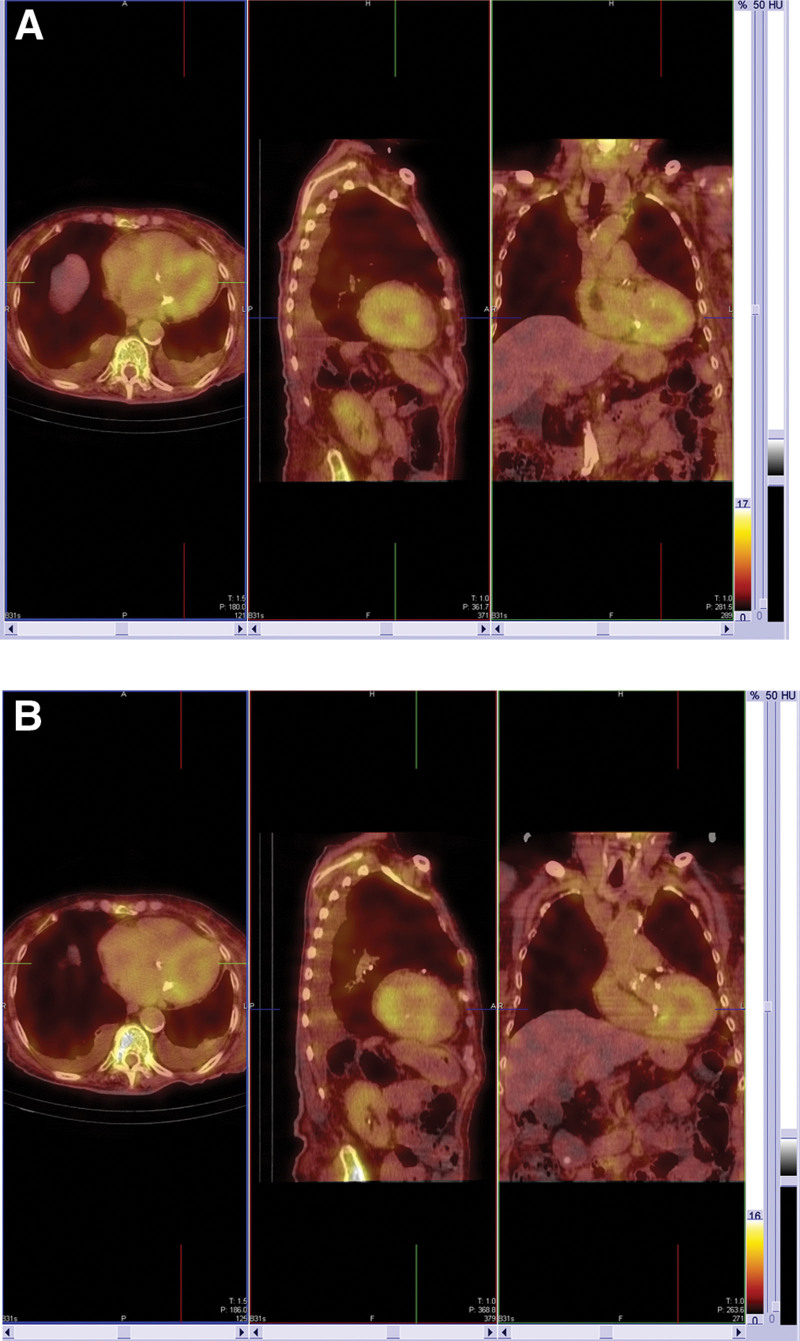
Reconstructions corrected for attenuation and scatter for SPECT/CT data for the same patient as Figures 1 and 2 for data acquired (A) 1 h following tracer injection and (B) 3 h after tracer injection. Both scans were scored as “3,” as the physician reading these studies perceived myocardial uptake to be greater than rib uptake and blood pool activity. The transaxial, saggital and coronal sections show SPECT data fused with CT images. CT = computed tomography, LV = left ventricle, SPECT = single photon emission computed tomography.

One-hour anterior and posterior planar images were acquired as 128 × 128 matrices with 750 × 10^3^ counts (Fig. 1). SPECT/CT images were acquired as 256 × 256 matrices for 128 projections for 35 seconds per projection. SPECT reconstructions by ordered subset expectation maximization (8 subsets, 3 iterations) were performed for this data, without attenuation correction, to emulate data that would be acquired on SPECT systems (Fig. 2). CT data were acquired immediately after SPECT acquisition using the CT acquisition parameters recommended by the manufacturer. Data were reconstructed using ordered subset expectation maximization (8 subsets, 3 iterations) and corrected for radiation scatter and attenuation using CT parameters and the reconstruction algorithm provided by the manufacturer (Fig. 3).

### 2.3. Planar image assessment

To generate planar HCL ratios, 1 physician manually drew a circular region of interest encompassing the perceived region of cardiac activity on the anterior planar image and then mirrored the same region to the contralateral side over the lung. Planar HCL ratios were classified using standard criteria: negative (HCL < 1.0), equivocal (HCL 1.0–1.5), or positive (HCL > 1.5) (Fig. 1).^[4]^

### 2.4. SPECT and SPECT/CT assessment

A different experienced Nuclear Medicine physician compared myocardial radiopharmaceutical uptake to rib uptake while viewing SPECT/CT fused image displays and grading uptake using the Perugini scale as revised in a recent ASNC consensus statement: 0 (no myocardial uptake), 1 (myocardial uptake < rib uptake), 2 (myocardial uptake = rib uptake), or 3 (myocardial uptake > rib uptake).^[6,8]^ On a separate occasion, the same individual compared myocardial radiopharmaceutical uptake to rib uptake while viewing SPECT images that included rotating maximum intensity projection cines along with multiple sections of transaxial, sagittal, and coronal tomographic projections. The same reader also subjectively graded SPECT/CT and SPECT image quality on a 3-point scale:1 (poor), 2 (adequate), or 3 (good).

To minimize potential bias, SPECT/CT and SPECT interpretations and image quality assessments were performed without reference to one another, quantitative results, or clinical patient information.

### 2.5. Statistical analysis

Statistical analysis was performed using MedCalc software (MedCalc Software Ltd., Ostend, Belgium).^[9]^ Values are reported as the mean ± 1 standard deviation. The normality of continuous variable distributions was determined using the *χ*^2^ test. Analysis of variance with Bonferroni correction was used to test the significance of differences in parameters among the visual score classes. The strength of the rank correlation of the parameters to the visual scores was assessed using Spearman *ρ*. Significance of differences was assessed using the unpaired or paired *t* test for normally distributed variables; otherwise, the Mann–Whitney or Wilcoxon test was used. Inter-rater agreement of visual scores was determined using the kappa statistic,^[10]^ for which the strength of agreement was considered “poor” for *κ* < .20, “fair” for *κ* = .21 − .40, “moderate” for *κ* = .41 − .60, “good” for *κ* = .61 − .80, and “very good” for *κ* ≥ .81. Proportions were compared using the *χ*^2^ test. Positive cases were defined by 3-hour SPECT/CT scores ≥ 2, negative cases as score = 0, and equivocal cases as score = 1. These were further dichotomized as negative for SPECT/CT visual scores < 2 and positive for scores ≥ 2, consistent with the analyses by previous investigators.^[11]^ The significance of the differences (Δ) between paired proportions in identifying positive cases was evaluated using McNemar test. Receiver operating characteristic analysis was used to determine the accuracy, sensitivity, and specificity of the other imaging modality readings in agreement with dichotomous 3-hour SPECT/CT readings. Test probability (*P*) < .05 was considered to be statistically significant, or as adjusted by Bonferroni corrections for comparisons among multiple categories.

## 3. Results

### 3.1. Data characterization

**Figure 4.**
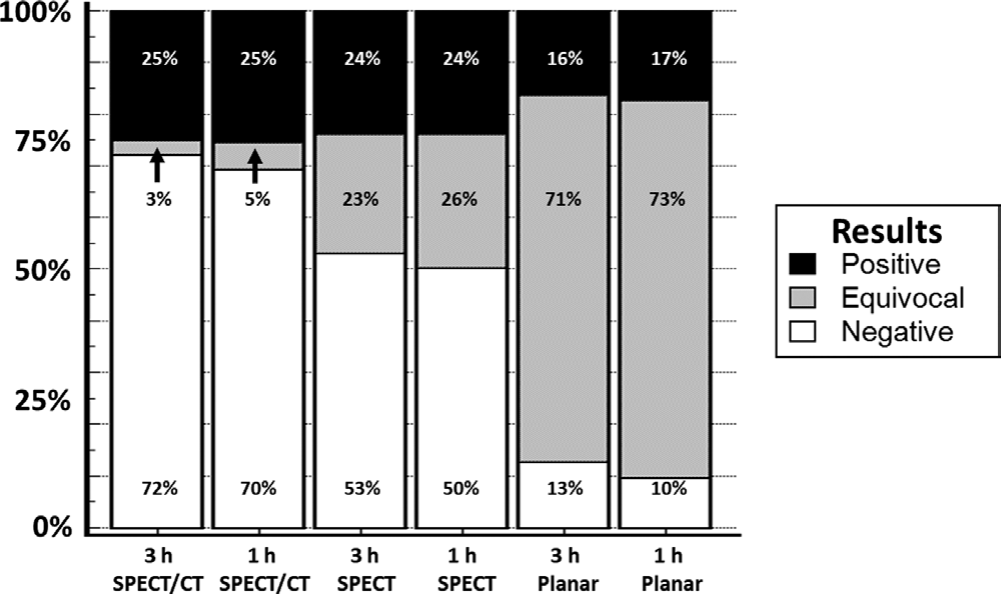
The percentage of cases found to be positive, equivocal, or negative are shown for the 6 imaging methods. The 3-h SPECT/CT method had the highest number of positive and negative cases and the lowest percentage (3%) of equivocal studies, while 1-h planar imaging for the same patient population had the lowest number of positive and negative cases and the highest percentage (73%) of equivocal studies. CT = computed tomography, SPECT = single photon emission computed tomography.

In our study population, males were younger than females (71 ± 12 vs 75 ± 11 years, *P* = .02). The composition of patient populations referred for imaging evolved over time, with 42% of cases positive in year 1 (N = 19), 26% in year 2 (N = 100), and 17% in year 3 (N = 54); therefore, the prevalence was significantly greater in the first year than in subsequent years (42% vs 17%, *P* = .03). In aggregate, 43 of the 173 patients were positive on 3-hour SPECT/CT, 125 were negative, and 5 were equivocal (Table 1; Fig. 4). Patients with positive results were older than the remaining patients in the cohort (80 ± 9 vs 70 ± 11 years, *P* < .001), with a similar prevalence among males and females (28% vs 20%, *P* = .25).

**Table 1:** One and 3-h SPECT/CT, SPECT, and planar case categorization.

	3-h SPECT/CT					
		0	1	2	3	% Cases
1-h SPECT/CT	0	116	4	0	0	70% (120/173)
	1	8	0	0	0	5% (9/173)
	2	1	1	0	1	1% (2/173)
	3	0	0	0	42	24% (42/173)
	% Cases	72% (125/173)	3% (5/173)	0% (0/173)	25% (43/175)	100%
	**3-h SPECT**					
		0	1	2	3	% cases
1-h SPECT	0	68	19	0	0	50% (87/173)
	1	24	20	0	1	26% (45/173)
	2	0	0	0	1	1% (1/173)
	3	0	1	2	37	23% (40/173)
	% Cases	53% (92/173)	23% (40/173)	1% (2/173)	23% (39/173)	100%
	**3-h Planar imaging**					
		Negative	Equivocal	Positive	% cases	
1-h Planar Imaging	Negative	14	3	0	10% (17/173)	
	Equivocal	8	116	2	73% (126/173)	
	Positive	0	4	26	17% (30/173)	
	% Cases	13% (22/173)	71% (123/173)	16% (28/173)	100%	

CT = computed tomography, SPECT = single photon emission computed tomography.

### 3.2. Comparison of 1-hour to 3-hour results

HCL ratios at 3 hours were slightly, but significantly, lower than at 1 hour (1.23 ± .24 vs 1.26 ± .25, *P* < .001). There was a similar percentage of positive results at both time points (Δ = 1.2%, *P* = .69). The percentage of equivocal cases was the same at 1 and 3 hours (73% vs 71%, *P* = .68). Of the cases that were equivocal at 1 hour, 92% (116/126) remained equivocal, 6% (8/126) were negative, and 2% (2/126) were positive at 3 hours (Table 1).

There was “good agreement” (*κ* = .76; *P* < .001) between the 1- and 3-hour SPECT scores (Table 1), with no difference in the number of positive cases. The percentage of equivocal cases was the same at 1 and 3 hours (26% vs 23%, *P* = .51). Of the cases that were equivocal at 1 hour, 44% (20/45) remained equivocal, 53% (24/45) were negative, and 2% (1/45) were positive at 3 hours (Table 1).

There was “very good agreement” (*κ* = .92, *P* < .001) between the 1 and 3 hours SPECT/CT scores (Table 1), with no significant difference in the number of positive cases (Δ = .6%, *P* = 1.00). The percentage of equivocal cases was the same at 1 and 3 hours (5% vs 3%, *P* = .34). None of the cases that were equivocal at 1 hour were equivocal at 3 hours, and all were negative (Table 1).

### 3.3. Comparison of SPECT/CT readings to SPECT readings and planar HCL ratios

Compared to the 3-hour SPECT/CT readings, there was “fair agreement” (*κ* = .27 − .33) with SPECT readings at 1 and 3 hours and “fair agreement” (*κ* = .23 − .31) with planar HCL values at 1 and 3 hours (Table 2). More patients were abnormal by dichotomized 3-hour SPECT/CT scores than by 1- or 3-hour planar HCL (25% vs 16–17%), and the difference was significant (Δ = 8–9%, *P* < .007). A similar proportion of patients identified as positive on 3-hour SPECT/CT was positive on 1- and 3-hour SPECT (Δ = 1–2%, *P* > .62).

**Table 2:** Three-hour SPECT/CT versus SPECT and planar imaging at 1 and 3 h.

		3-h SPECT/CT				
		0	1	2	3	% Cases
3-h SPECT	0	91	1	0	0	53% (92/173)
	1	33	4	0	3	23% (40/173)
	2	0	0	0	2	1% (2/173)
	3	1	0	0	38	23% (39/173)
1-h SPECT	0	82	4	0	1	50% (87/173)
	1	43	1	0	1	26% (45/173)
	2	0	0	0	1	1% (1/173)
	3	0	0	0	40	23% (40/173)
3-h Planar imaging	Negative	22	0	0	0	13% (22/173)
	Equivocal	102	5	0	16	71% (123/173)
	Positive	1	0	0	27	16% (28/173)
1-h Planar imaging	Negative	17	0	0	0	10% (17/173)
	Equivocal	104	5	0	17	73% (126/173)
	Positive	4	0	0	26	17% (30/173)
	% Cases	72% (125/173)	3% (5/173)	0% (0/173)	25% (43/173)	100%

CT = computed tomography, SPECT = single photon emission computed tomography.

The percentage of equivocal cases was higher on planar 1- and 3-hour HCL values than on 1- and 3-hour SPECT (71–73% vs 23–26%, *P* < .001), and 1- and 3-hour SPECT/CT (3–5%, *P* < .001). Cases that were equivocal on planar HCL was significantly more negative than positive on 1- and 3-hour SPECT (58–62% vs 12%, *P* < .001), and on 1 and 3-hour SPECT/CT (83% vs 13%, *P* < .001) (Table 2; Fig. 4). Using 3-hour SPECT/CT readings as the reference standard, 1-hour HCL and 3-hour HCL criteria were significantly less accurate than SPECT and 1-hour SPECT/CT (86–88% vs 98–99%, *P* < .001) (Table 3).

**Table 3:** Receiver operating characteristic results using 3-h SPECT/CT as the reference standard.

Method	ROC threshold	AUC (N = 166)	Accuracy (N = 175)	Sensitivity (N = 44)	Specificity (N = 131)	PPV	NPV
1-h SPECT/CT	>1	99 ± 1%	98%	98%	98%	96%	99%
3-h SPECT	>1	99 ± 1%	98%	93%	99%	98%	98%
1-h SPECT	>1	98 ± 2%	99%	95%	100%	100%	99%
3-h Planar	> 1.22	95 ± 2%*	86%*	95%	83%*	66%*	98%
1-h Planar	> 1.32	94 ± 2%*	88%*	89%	88%*	71%*	96%

AUC = ROC area under the curve, CT = computed tomography, NPV = negative predictive value, PPV = positive predictive value, ROC = receiver operating characteristic, SPECT = single photon emission computed tomography.

* *P* < .05 vs 1-h SPECT/CT.

### 3.4. Image quality

At both 1 and 3 hours, the image quality scores were higher for SPECT/CT than for SPECT (2.7 ± .5 vs 2.1 ± .5, *P* < .001; Table 4). Three-hour SPECT/CT image quality score was higher than the 1-hour SPECT/CT image quality score (2.8 ± .4 vs 2.5 ± .6, *P* < .001).

**Table 4:** Image quality.

	Image quality
3-h SPECT/CT	2.8 ± .4
1-h SPECT/CT	2.5 ± .6*
3-h SPECT	2.1 ± .5*^,^†
1-h SPECT	2.0 ± .5*^,^†

CT = computed tomography, SPECT = single photon emission computed tomography.

* *P* < .05 vs 3-h SPECT/CT.

† *P* < .05 vs 1-h SPECT/CT.

## 4. Discussion

Radionuclide imaging with ^99m^Tc-PYP was first used in cardiac diagnosis in the 1970s to evaluate patients with myocardial infarction. Parkey and Willerson showed that calcification in the peripheral remodeling zone of myocardial infarcts binds PYP and that this label could be used as a marker of myocardial infarction.^[12]^ Cardiac uptake of PYP has been reported in patients with congestive heart failure (HF) and amyloid cardiomyopathy.^[13,14]^ However, the relationship between congestive HF and amyloids is unreliable and inconsistent. These early studies preceded recognition of the pathogenesis of clinical subtypes of amyloidosis and the establishment of transthyretin deposition as the cause of what was then referred to as “senile” amyloidosis.^[15]^

The use of bone-avid agents to diagnose cardiac amyloid was reintroduced in 2005 by Perugini et al,^[8]^ who employed the bone agent ^99m^Tc-DPD to evaluate patients with cardiac amyloidosis. A scale of 0–3 was used for grading myocardial uptake of radiopharmaceutical. They were able to separate 15 patients with known ATTR from 10 patients with AL with 100% accuracy using the criterion of > 2 uptakes for ATTR. A larger collaborative study drawn from amyloidosis clinics in the US, UK, and Europe, the “Multicenter Trial of Diphosphonates in the Diagnosis of Amyloidosis” in 1217 patients (including 374 with biopsy-proven disease) yielded a sensitivity of 100% and specificity of 86% using Perugini criteria.^[1]^

In the US, only ^99m^Tc-PYP is available. This agent has been studied by Bokhari et al in 45 patients with known amyloidosis.^[4]^ They quantified myocardial uptake using a planar HCL uptake ratio, for which a value >1.5 was 97% sensitive and 100% specific for ATTR cardiac amyloidosis. These findings were confirmed by Castano et al, who used ^99m^Tc-PYP to study 171 patients, including 155 with proven ATTR (N = 121) or AL (N = 34) amyloid.^[2]^ The HCL ratio achieved 91% sensitivity and 92% specificity for ATTR. Based on these studies, current guidelines suggest that planar ^99m^Tc-PYP studies can be used accurately and noninvasively to diagnose ATTR cardiac amyloid once light chain disease has been excluded.^[16]^

The above data were compiled predominantly from amyloid centers of investigation. ^99m^Tc-PYP scintigraphy has also been used in other clinical settings. Patients with aortic stenosis^[17]^ and diastolic HF^[3]^ have been found to have positive scans and ATTR amyloidosis. ^99m^Tc-PYP imaging has been widely utilized as a diagnostic technique to investigate patients with HF, particularly in the setting of left ventricular wall thickening, arrhythmias, and conduction abnormalities.^[16]^ However, as has occurred with other nuclear cardiology techniques such as myocardial perfusion imaging, as referral categories become more broad-based, the specificity of findings and the number of borderline or non-diagnostic cases may increase.^[18,19]^

The effect of referral bias is also evident in our data. Over 3 years, the percentage of positive cases declined from 42 to 17%. A likely explanation for this trend is that initially patients who were referred for evaluation were those who had been preselected to have positive biopsy evidence or DNA evidence of ATTR,^[4]^ but following the issuance of a series of imaging guidelines by professional societies, referring physicians cast a wider net due to the realization that ATTR as a cause of HF had been under-recognized.^[16]^ Current guidelines suggest that patients be evaluated for possible amyloidosis who have unexplained HF and increased left ventricular wall thickness. These guidelines list a wide range of indications, with recommended patient selection criteria for amyloidosis evaluation having now become rather nonspecific. Listed indications include African-Americans over 60 years with HF, patients over 60 years with unexplained HF with preserved ejection fraction, elderly males with unexplained neuropathy, bilateral carpal tunnel syndrome or atrial arrhythmias in the absence of usual risk factors, and known or suspected familial amyloidosis. As there are other underlying conditions that can produce some of these same symptoms, it is not surprising that ATTR prevalence has fallen among unselected patient populations.

Our high planar equivocal rate is similar to a study of 122 patients by Regis et al,^[5]^ who found that 66% of patients fell into the range termed “non-diagnostic” by 1-hour planar HCL but only 8% from 3-hour SPECT/CT visual assessment, similar to our overall results of 73% and 3%, respectively (Table 1). The similarities we found between the 1-hour and 3-hour planar case categorizations (Table 1) were also found in an investigation of 109 patients by Sperry et al in a protocol optimization investigation.^[20]^

Repeating a 1-hour planar study because it was non-diagnostic by acquiring a 3-hour planar study is not useful; 92% of the cases in our study that were equivocal at 1 hour remained equivocal at 3 hours. As pointed out by Bokhari and Cerqueira, imaging institutions have a variety of options in sorting through the possibility of evaluating cardiac amyloidosis, and despite studies to address the optimization of protocols, a definitive conclusion has yet to be reached.^[21]^ In a freestanding clinical setting that is not an amyloid center, one can expect a large percentage of equivocal cases. We documented that over 70% of cases are equivocal on planar imaging, and we found that 3-hour planar imaging is of negligible help in reducing the number of equivocal cases at 1 hour.

We found negligible differences in the identification of positive cases between 1-hour and 3-hour evaluations by planar imaging, SPECT, and SPECT/CT. Consequently, we conclude that there is no advantage in dual-time-point ATTR imaging with ^99m^Tc-PYP, regardless of the imaging technique. This result has the practical advantage of enabling flexibility in the timing of the imaging protocol implementation.

A reasonable question to ask is whether SPECT/CT technology is necessary for cardiac amyloidosis evaluation, or whether the more widely available SPECT technology is sufficient. In our study, while SPECT evaluations had the advantage over planar HCL criteria of producing a significantly lower percentage of equivocal results (23–26% vs 71–73%), SPECT/CT eliminated nearly all equivocal results with only 3 to 5% equivocal cases. In addition, image quality was judged to be the highest for 3-hour SPECT/CT, significantly higher than 1-hour SPECT/CT and 1- and 3-hour SPECT (Table 4), while sensitivity to detect positive cases was 93% for SPECT compared to SPECT/CT (Table 3). We attribute both observations to the fact that SPECT-only images are not corrected for attenuation, and because the fusion of the CT with the SPECT emission data allows the reader to distinguish myocardial uptake from blood pool and skeletal (rib, sternum) ^99m^Tc-PYP uptake. Confusion as to whether tracer uptake is due to blood pool, skeletal or myocardial uptake is also the reason that planar HCL ratios are less successful in accurately identifying patients with ATTR. Therefore, 3-hour SPECT/CT is clearly preferable to SPECT and planar imaging for diagnosing ATTR and is the only scan needed in evaluating the possibility of cardiac amyloidosis in an unselected patient population.

Given our findings, it is hoped that, when available, SPECT/CT, rather than planar imaging and SPECT, will be performed for patients being evaluated for possible cardiac amyloidosis. Furthermore, we hope that our investigation will stimulate imaging laboratories to acquire SPECT/CT technology if they do not already have this modality available.

### 4.1. Study limitations

Our investigation sought to determine whether the same or different categorizations of patient results were obtained at different time points by SPECT/CT, SPECT, or planar imaging, starting with 3-hour SPECT/CT as the reference standard. We did not have other diagnostic imaging test results (e.g., echocardiography or cardiac magnetic resonance imaging^[1]^) or biopsy information against which to refer as independent determinants of cardiac ATTR. Consequently, our rationale in using 3-hour SPECT/CT as the reference standard is that 3-hour imaging should, in general, be more successful at revealing ATTR than 1-hour imaging due to blood clearance with time,^[20]^ and because the localization of activity with the CT unit should aid in distinguishing myocardial from blood pool and skeletal activity.^[5]^

## 5. Conclusion

While there is good agreement between time points for SPECT/CT, SPECT, and planar modalities individually, 3-hour SPECT/CT readings provided the highest number of definitive readings, had the highest image quality, and constituted the preferred protocol for evaluating unselected populations of patients that have a clinical suspicion of cardiac amyloidosis.

## Author contributions

**Conceptualization:** Kenneth J. Nichols, Andrew Van Tosh, Christopher J. Palestro.

**Data curation:** Kenneth J. Nichols, Se-Young Yoon.

**Formal analysis:** Kenneth J. Nichols, Se-Young Yoon.

**Project administration:** Kenneth J. Nichols, Christopher J. Palestro.

**Visualization:** Se-Young Yoon, Christopher J. Palestro.

**Writing – original draft:** Kenneth J. Nichols, Se-Young Yoon, Andrew Van Tosh, Christopher J. Palestro.
